# *Neooleiphilus portus* gen. nov., sp. nov. and *Paroleiphilus aggregans* gen. nov., sp. nov., Two Novel Nitrate-Reducing Bacteria Isolated from Estuarine Sediments

**DOI:** 10.3390/microorganisms14102307

**Published:** 2026-10-10

**Authors:** Zhaobin Huang, Qiliang Lai

**Affiliations:** 1 College of Oceanology and Food Science, Quanzhou Normal University, Quanzhou 362000, China; 2 Fujian Province Key Laboratory for the Development of Bioactive Material from Marine Algae, Quanzhou 362000, China; 3 Key Laboratory of Marine Genetic Resources, Third Institute of Oceanography, Ministry of Natural Resources, Xiamen 361005, China; lai719@163.com

**Keywords:** estuarine sediment, *Neooleiphilus portus*, *Paroleiphilus aggregans*, denitrification, polyphasic taxonomy

## Abstract

Two novel bacteria, designated strain G1-8^T^ and J1-3^T^, were isolated from estuarine sediment samples collected from Jinjiang Estuary, Fujian Province, China, using the dilution-to-extinction method. Cells of strain G1-8^T^ were Gram-stain-negative, aerobic, rod-shaped with a single polar flagellum, and had a surface layer. Cells of strain J1-3^T^ were also Gram-stain-negative, aerobic, and short rod-shaped, yet the flagellum and the surface layer were not observed. Reduction of nitrate to nitrite under aerobic conditions was positive for both stains. Strains G1-8^T^ and J1-3^T^ shared the highest 16S rRNA gene sequence similarities with *Oleiphilus messinensis* ME102^T^ of 94.45% and 93.77%, respectively. Phylogenetic analysis placed the two strains into two different monophyletic lineages, affiliated with the family *Oleiphilaceae* of the class *Gammproteobacteria*. The digital DNA-DNA hybridization, average nucleotide identity, and average amino acid identity between the two strains and *O. messinensis* ME102^T^ were 21.2–22.6%, 68.6–68.8%, and 58.2–59.3%, respectively. The major fatty acids (>10%) of strain G1-8^T^ were C_16:0_ (24.9%), C_16:1_ *ω*9*c* (14.9%), C_18:1_ *ω*9*c* (14.2%), and summed feature 3 (13.1%), and the major fatty acids (>10%) of strain J1-3^T^ were summed feature 3 (39.6%) and C_16:0_ (37.0%). The isoprenoid quinone of both strains was Q-9. The polar lipids of strain G1-8^T^ consisted of phosphatidylglycerol and two unidentified phospholipids, and the polar lipids of strain J1-3^T^ consisted of phosphatidylethanolamine and two unidentified lipids. Based on the above polyphasic taxonomic study, strains G1-8^T^ and J1-3^T^ were considered to represent two novel species within two novel genera of the family *Oleiphilaceae*. Thus, two species names were proposed: *Neooleiphilus portus* gen. nov., sp. nov., with the type strain G1-8^T^ (=MCCC 1K10098^T^=KCTC 18326^T^), and *Paroleiphilus aggregans* gen. nov., sp. nov., with the type strain J1-3^T^ (=MCCC 1K10092^T^=KCTC 18325^T^).

## 1. Introduction

Estuaries are among the most productive ecosystems on Earth, serving as a transition between terrestrial and marine environments [1], which are largely influenced by climate and anthropogenic pollutants, such as oil [2] and heavy metals [3]. Microbes in estuarine sediments play crucial roles in nutrient breakdown and biogeochemical cycles, and can respond swiftly to human activities [4]. However, the majority of microbes remain uncultivated, which restricts our understanding of their physiological and metabolic processes, and further hinders the development of biotechnological applications [5].

Bacteria of the family *Oleiphilaceae*, affiliated with the class *Gammaproteobacteria*, have been found in diverse marine environments [6]. Based on the List of Prokaryotic names with Standing in Nomenclature (LPSN) records, the family *Oleiphilaceae* includes only one genus and only one species, *Oleiphilus messinensis*, thus far [7]. Much attention has been devoted to *O. messinensis*, due to its obligate alkane-degrading capability, which allows it to utilize aliphatic hydrocarbons and their derivatives as the sole source of carbon and energy [7,8].

In this study, two novel bacteria, designated strains G1-8^T^ and J1-3^T^, were obtained from estuarine sediment samples collected from Jinjiang Estuary, Quanzhou City, Fujian Province, China, using the dilution-to-extinction method. Preliminary analysis of the 16S rRNA gene sequences of the two strains showed that they were affiliated with the family *Oleiphilaceae*, and had a maximum sequence similarity of 94.5% with the validly published species and the uncultivated bacteria of the core nucleotide BLAST v.2.17.0 database in the NCBI (National Center for Biotechnology Information), indicating that they may represent novel species within novel genera [9]. Therefore, we conducted a polyphasic taxonomic characterization to confirm the taxonomic assignment of the two strains.

## 2. Materials and Methods

### 2.1. Bacterial Isolation and Cultivation

Strains G1-8^T^ and J1-3^T^ were isolated from two different sampling sites, named G (24.884698 N, 118.585757 E) and J (24.879878 N, 118.618731 E), respectively, alongside the Jinjiang Estuary coast in Quanzhou City, Fujian Province, China, on 21 April 2025 (Figure S1). The salinity at site G and site J was measured to be 12‰ and 18‰, respectively, using a portable refractometer (LH-Y100, Lohang Bio. Co., Hangzhou, China). The surface sediment was collected using a sterile 60 mL syringe on the ebb tide. The sediment at a depth of 5 cm was then cut and collected using a sterile blade; 1 g sediment sample was dissolved in 9 mL of sterile seawater, and then subjected to serial dilution (10^−1^–10^−4^). Strains were isolated from the 10^−4^ dilution sample using the dilution-to-extinction method. Briefly, 1 μL was inoculated into each well of the 96-well plate (Haier Biomedical product, Qingdao, China) containing 180 μL 2216E culture medium (5 g/L peptone, 1 g/L yeast extract, 1 L natural seawater). The plates were placed in an incubator at 30 °C for 7 days. An aliquot of 10 uL of the culture liquid was taken and streaked onto 2216E agar plates to obtain the pure culture. Strains G1-8^T^ and J1-3^T^ were finally obtained and preserved in 20% glycerol (*v*/*v*).

### 2.2. Phylogeny of 16S rRNA Gene

The genomic DNA of strains G1-8^T^ and J1-3^T^ was obtained using heat lysis at 99 °C for 10 min. The 16S rRNA gene sequence was then PCR-amplified using universal primers Eubac27F and 1492R [10] in a 50 μL amplification system (*Ex* Taq, TaKaRa). The partial sequence was determined using an ABI 3730 sequencer (Shanghai Sangon Biotech Co., Ltd.) with the universal primers, and assembled into a nearly full-length sequence using DNAMAN v 8.0. The closest relatives were identified using EzBioCloud’s identification service [11], and NCBI nucleotide blast (https://blast.ncbi.nlm.nih.gov/Blast.cgi, accessed on 14 July 2025). Phylogenetic analysis was performed by following our previous study [12].

### 2.3. Genomic Sequencing and Analysis

The genomic DNA of strains G1-8^T^ and J1-3^T^ was extracted from the fresh cells using a bacterial DNA extraction kit (Shanghai SaiBaisheng Biotech. Co., Shanghai, China), and then was used for the whole-genome sequencing using the Illumina Hiseq platform (Shanghai Majorbio Bio-Pharm Technology Co., Ltd., Shanghai, China). The PE reads were quality-checked using sickle (https://github.com/najoshi/sickle, accessed on 17 July 2025), and assembled into contiguous sequences (contigs) using SPAdes v. 3.8.1 with *k* values of 21, 33, 55, 77, 99, 127 and -*careful* option [13].

The 16S-23S-5S rRNA gene sequences (*rrn* operon) of strains G1-8^T^ and J1-3^T^ were identified and extracted from the whole-genome sequence using RNAmmer [14], and then the 16S rRNA gene sequence was compared with that obtained by the PCR-amplified sequence described above. Functional genes were predicted using Prodigal [15], and annotated using eggnogmapper [16], Protologger [17], and KAAS [18].

The digital DNA-DNA hybridization (dDDH) values were calculated using the genome-to-genome distance calculator 3.0 (GGDC 3.0) [19], and the average nucleotide identity (ANI) values were calculated using the ANI calculator on the EzBioCloud website [20].

### 2.4. Phylogenomic Analysis

The genomes of type strains closely related to the strains 1-8^T^ and J1-3^T^ were downloaded from the Genome dataset in GenBank (https://www.ncbi.nlm.nih.gov/datasets/genome/?taxon=135619, accessed on 14 July 2025). The genomes of the uncultivated bacteria were downloaded from GenBank (accessed on 14 July 2025) and used for genomic comparison. Genome quality was checked using CheckM version 1.2.0 [21], and the genomes with >90% completeness and <5% contamination were included in the following analysis. The phylogenomic tree was constructed using GTDB-Tk version 1.3.0 [22]. The average amino acid identity (AAI) values were calculated using the AAI calculator in Enveomics Gateway online (https://enveomics.scigap.org/, accessed on 17 July 2025) and CompareM (https://github.com/donovan-h-parks/CompareM, accessed on 17 July 2025).

### 2.5. Phenotypic Characterization

Gram staining, cell morphology, activities of catalase and oxidase were tested as previously described [12]. Motility was tested using the puncture inoculation into the 2216E semi-solid agar (0.5%, *w*/*v*) medium. The growth temperature was tested by inoculating the cells at 15–45 °C (at intervals of 5 °C). The NaCl tolerance was tested by inoculating the cells with 0, 0.5, 1–12.0% (in increments of 1%, *w*/*v*) using a modified 2216E medium [23]. The pH range was tested by inoculating the cells in 2216E medium adjusted to pH 4.0–6.0 (Na_2_HPO_4_-citrate acid solution) and pH 7.0–9.0 (Tris-HCl solution). Reduction of nitrate to N_2_ under aerobic conditions was tested in 2216E medium supplemented with 0.1% (*w*/*v*) KNO_3_, using a 15 mL tube equipped with an inverted Durham tube for monitoring the bubble production. Anaerobic growth was tested in 2216E supplemented with 0.1% (*w*/*v*) KNO_3_, 0.05% (*w*/*v*) L-cysteine hydrochloride and 0.001% (*w*/*v*) resazurin in Hungate tubes, prepared under 100% N_2_ gas. The strains were also inoculated into the 100 mL ONR7a medium (https://www.bacmedia.dsmz.de/medium/950, accessed on 17 July 2025) containing 0.1% hexadecane (*v*/*v*) and 0.1% pentadecane (*v*/*v*) to test the ability to degrade hydrocarbon. The other physiological and biochemical tests were evaluated using API ZYM, API 20NE and API 20E strips (bioMérieux China Limited product, Guangzhou, China) according to the manufacturer’s instructions.

### 2.6. Chemotaxonomic Characterization

The cellular fatty acids, respiratory quinones and polar lipids of strains G1-8^T^ and J1-3^T^ were extracted and identified by following the previously published studies [12,24,25].

## 3. Results and Discussion

### 3.1. Phylogeny of 16S rRNA Gene

The lengths of PCR-amplified 16S rRNA gene sequences of strains G1-8^T^ and J1-3^T^ were 1394 bp and 1390 bp, respectively. Strains G1-8^T^ and J1-3^T^ had the highest sequence similarities with *O. messinensis* ME102^T^ of 94.45% and 93.77%, respectively. These values were significantly below the 98.65% threshold established for species delineation [26], indicating that the two strains may represent a novel species. By conducting a 16S rRNA sequence similarity search, the PCR-amplified 16S rRNA gene sequences of strains G1-8^T^ and J1-3^T^ were found to have similarities of <94.5% with the uncultivated bacteria, including the mesopelagic *Oleiphilus*-related bacteria enriched with the high-molecular-weight dissolved organic matter (HMWDOM). These mesopelagic bacteria were reported to be abundant in the mesopelagic water in the ocean [27]. Therefore, the 16S rRNA gene sequence similarity search indicated that the PCR-amplified 16S rRNA gene sequences of strains G1-8^T^ and J1-3^T^ represented potential novel bacteria, which were not closely related to the mesopelagic bacteria.

**Figure 1:**
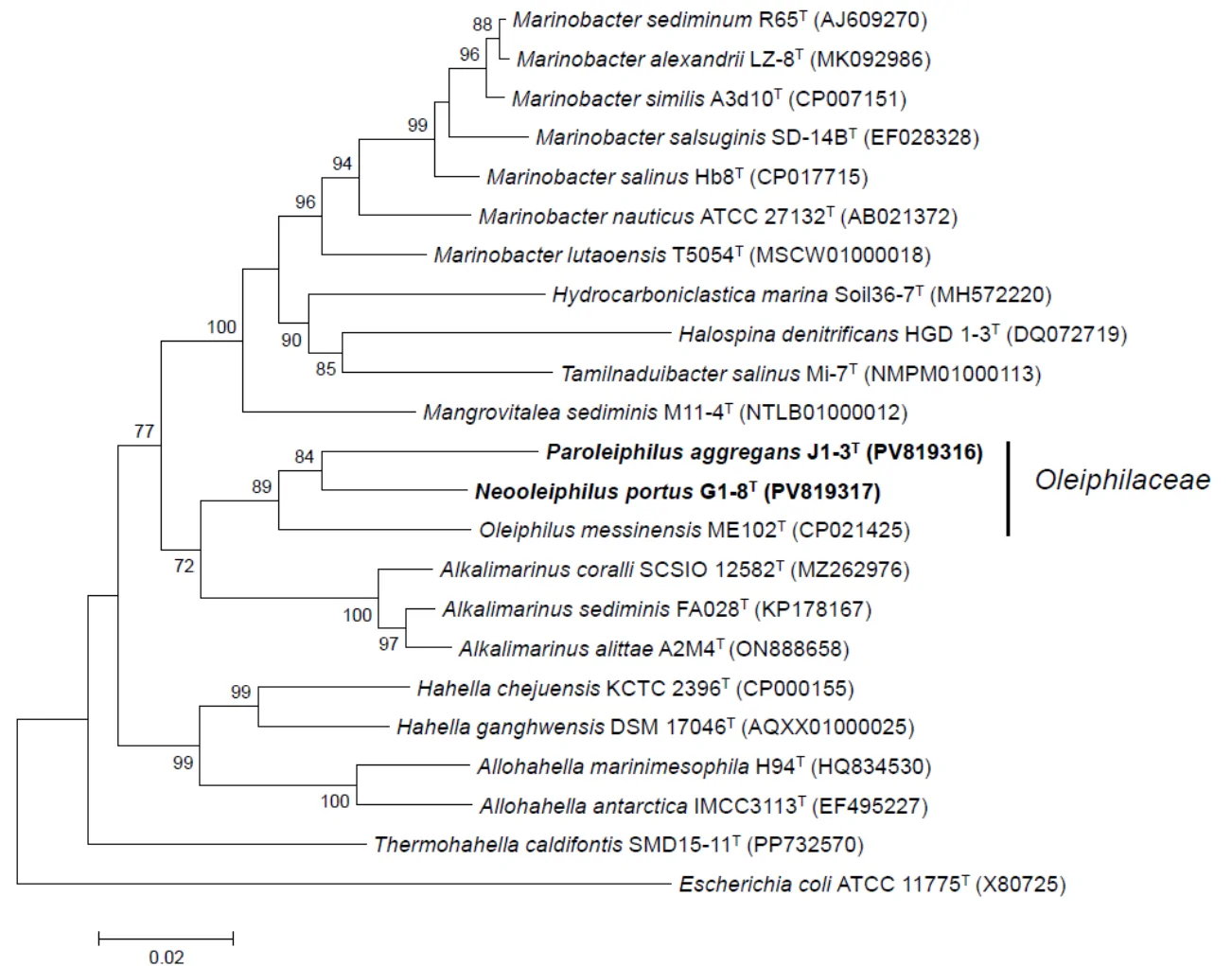
Phylogenetic tree constructed using the neighbor-joining method with 16S rRNA gene sequences showing the relationship between strains G1-8^T^ and J1-3^T^ and their close relatives. The node values >70% are shown on the branch. Bar, the nucleotide substitution.

The phylogenetic trees constructed using NJ and ML based on 16S rRNA gene sequences indicated that strains G1-8^T^ and J1-3^T^ clustered into two distinct monophyletic lineages, separate from *O. messinensis* ME102^T^ (Figure 1 and Figure S2).

### 3.2. Genomic Characterization

The genome size of strain G1-8^T^ was 4,948,536 bp on 35 contigs (>1000 bp in length), and genome completeness and contamination were estimated to be 98.3% and 1.2%, respectively. The genome size of strain J1-3^T^ was 4,888,805 bp on 63 contigs (>1000 bp in length), and genome completeness and contamination were estimated to be 99.4% and 1.4%, respectively. Both genomes had a sequencing coverage of >200X. Both genomes were much smaller than that of the *O. messinensis* strain ME102^T^ with 6,379,281 bp on one contig (a complete genome). The genomic G+C contents of strain G1-8^T^ and strain J1-3^T^ were 52.2% and 46.8%, respectively, which were also different from the *O. messinensis* strain ME102^T^ (Table 1). Based on the value of Formula 2 as recommended, the dDDH values of strains G1-8^T^ and J1-3^T^ compared to *O. messinensis* ME102^T^ were 22.6% and 21.2%, respectively. The dDDH value compared between strain G1-8^T^ and strain J1-3^T^ was 17.9% (Table S1), which was significantly below the threshold of species delineation (70%) [19]. The ANI values of strains G1-8^T^ and J1-3^T^ compared to *O. messinensis* ME102^T^ were 68.6 and 68.8%, respectively (Table S1). The ANI value compared between strains G1-8^T^ and J1-3^T^ was 68.2%. The ANI values were also significantly below the threshold values of the recommended species delineation (95–96%) [28].

Gene annotation found that strain G1-8^T^ possessed a nitrate reductase operon, *nar*KGHJI (Table S2), which encoded a membrane-bound nitrate reductase involved in the denitrification process [29]. Strain J1-3^T^ also possessed a gene cluster for denitrification, the *nap*DAGHB (Table S2), which encoded a periplasmic nitrate reductase. Similar to strain J1-3^T^, *O. messinensis* ME102^T^ had the *nap*DAB genes. The predicted denitrification activity was then confirmed by the API 20NE strip test, indicating that they may play an important role in nitrogen cycling of estuarine sediments.

### 3.3. Phylogenomic Analysis

**Figure 2:**
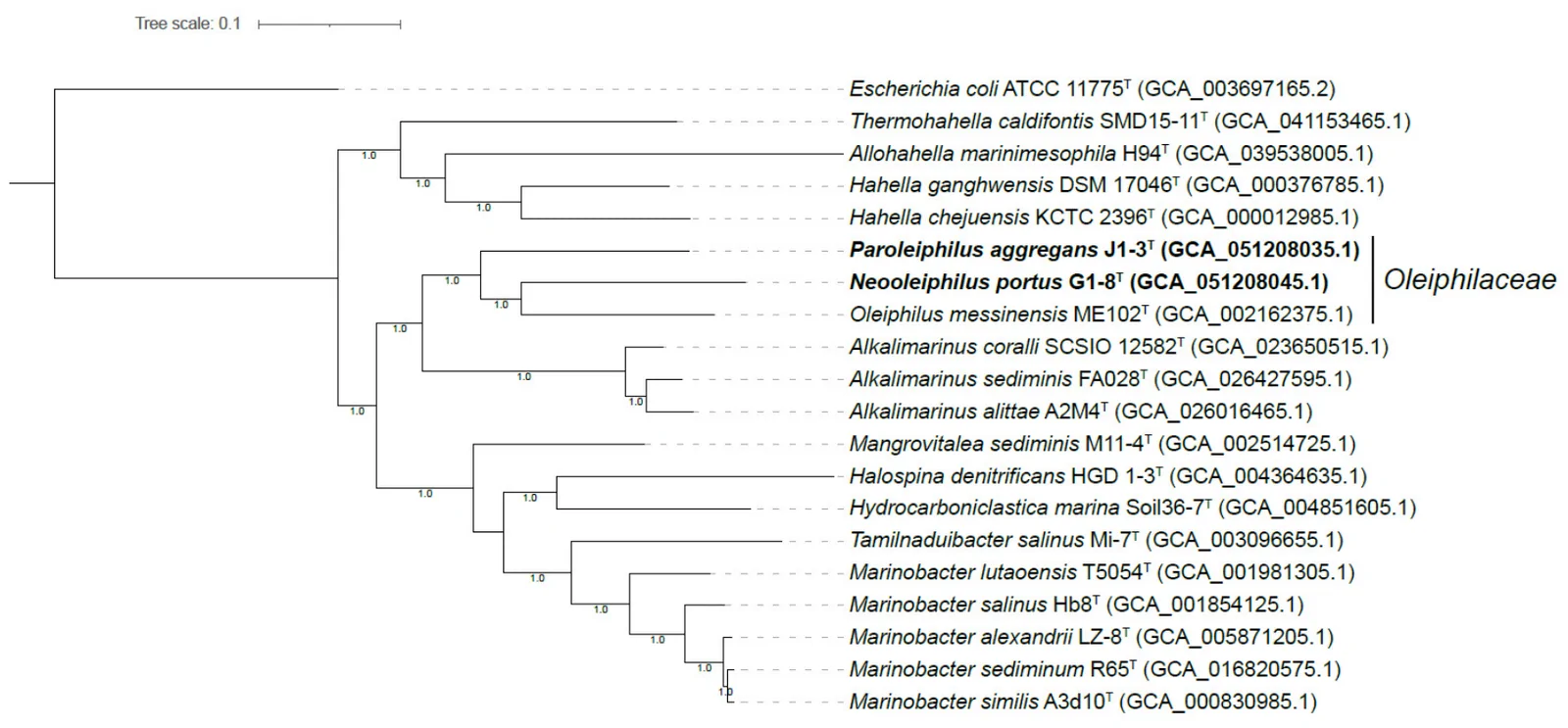
Phylogenomic tree constructed based on 120 bacterial conserved concatenated proteins showing the relationship of strains G1-8^T^ and J1-3^T^ and their close relatives. The bootstrap values of the node greater than 0.7 are displayed. Bar, 0.1, the amino acid substitution per position.

The phylogenomic tree clearly placed strains G1-8^T^ and J1-3^T^ into two novel distinct monophyletic lineages, which were closely related to *O. messinensis* ME102^T^ (Figure 2). strain J1-3^T^ was found to have a close relationship with a metagenome-assembled genome (MAG) named HK-STAS-PROT-159 [30], strain J1-3^T^ and MAG HK-STAS-PROT-159 shared the values of dDDH, ANI and AAI of 66.9%, 96.3%, and 96.5% respectively. The ANI and AAI values indicated that the MAG HK-STAS-PROT-159 could be considered the same species as strain J1-3^T^. The AAI values of strains G1-8^T^ and J1-3^T^ compared to *O*. *messinensis* ME102^T^ were 58.2 and 59.3%, respectively, which were also below the threshold values of recommended genus delineation (60–80%) [31]. A heatmap based on AAI values clearly placed strains G1-8^T^ and J1-3^T^ into two distinct groups, separate from *O. messinensis* ME102^T^ (Figure S3). Furthermore, we examined the ecological habitat of these bacteria closely related to *O. messinensis* ME102^T^ based on the recorded information of these genomes in GenBank. We found that the *O. messinensis*-related bacteria are all detected from marine environments, such as marine sediment and marine algae (Figure S3), indicating that they may be specialized in the marine environment.

### 3.4. Phenotypic Characterization

**Figure 3:**
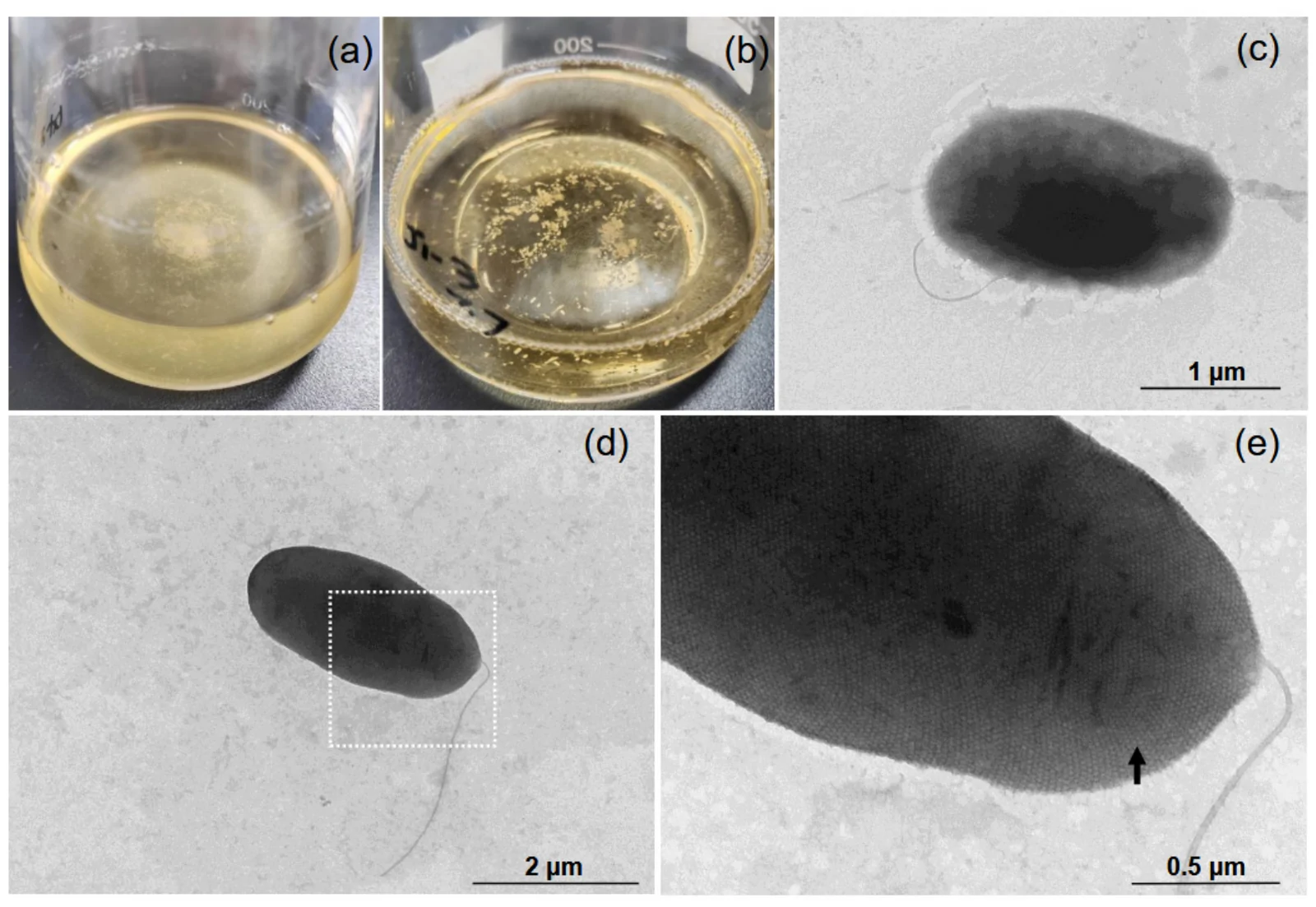
Growth in liquid medium and cell morphology observed under the TEM. (**a**,**b**), the growth of strains G1-8^T^ and J1-3^T^ in 2216E liquid medium, respectively. (**c**,**d**), the cellular morphology of strains G1-8^T^ and J1-3^T^, respectively, observed under the TEM. (**e**) Ultrastructural observation of strain G1-8^T^ and an S-layer structure (arrowhead).

Both strain G1-8^T^ and strain J1-3^T^ grew slowly on the 2216E agar plates, and small colonies (0.1 cm for G1-8^T^ and 0.05 cm for J1-3^T^ in diameter) were observed after cultivation for 7 days. Cells of strain G1-8^T^ can aggregate when grown in liquid medium (Figure 3). Cells of strain J1-3^T^ can also aggregate in liquid medium (Figure 3). Cells of strain G1-8^T^ were Gram-negative, aerobic, and rod-shaped, 1.6–2.9 μm in length and 0.9–1.5 μm in width, and a single polar flagellum and a surface layer (S-layer) was observed (Figure 3). Cells of strain J1-3^T^ were also Gram-negative, aerobic, and short rod-shaped, 1.7–2.2 μm in length and 1.0–1.3 μm in width, yet the flagellum and the S-layer were not observed (Figure 3). Cytochrome *c* oxidase activity of the two strains was positive, and catalase activity was negative. Growth of the two strains was observed at 25–40 °C with the optimum of 30 °C, NaCl 0–4.0% with the optimum of 1.0–2.0%, and pH 6.0–8.0 with the optimum of 7.0. Nitrate could be reduced to nitrite under aerobic conditions for strain G1-8^T^, which was weakly reduced for strain J1-3^T^. Denitrification from nitrate to N_2_ was not observed for both strains. Growth was not observed for strains G1-8^T^ and J1-3^T^ with 0.1% hexadecane and 0.1% pentadecane as sole carbon sources. The other detailed physiological and biochemical properties tested using the API strips are described in Table 1 and in the species description.

### 3.5. Chemotaxonomic Characterization

The fatty acid compositions compared among strain G1-8^T^, strain J-3^T^ and *O. messinensis* ME102^T^ showed quite different profiles (Table 2). The major fatty acids (>10%) of strain G1-8^T^ were C_16:0_ (24.9%), C_16:1_ *ω*9*c* (14.9%), C_18:1_ *ω*9*c* (14.2%), and summed feature 3 (13.1%). The major fatty acids (>10%) of strain J1-3^T^ were summed feature 3 (39.6%) and C_16:0_ (37.0%) (Table 2). The major fatty acid of *O. messinensis* ME102^T^ included C_16:0_ (38.6%), C_16:1_ *ω*9*c* (10.9%), and summed feature 3 (16.6%) [7]. C_16:1_ *ω*9*c* was a major fatty acid in strain G1-8 and *O. messinensis* ME102^T^, while it was not found in strain J1-3^T^. The major isoprenoid quinone of strain G1-8^T^ and strain J1-3^T^ was Q-9, which was the major isoprenoid quinone of the family *Oleiphilaceae* [32]. The polar lipids of strain G1-8^T^ included phosphatidylglycerol (PG) and two phospholipids (PLs), and the polar lipids of strain J1-3^T^ included phosphatidylethanolamine (PE) and two unidentified lipids (L) (Figure S4). The polar lipids of strains G1-8^T^ and J1-3^T^ showed different profiles with *O. messinensis* ME102^T^, which included phosphatidylglycerol (PG), phosphatidylethanolamine (PE), and phosphatidyl dimethylethylamine (DME) [7].

## 4. Conclusions

Two novel bacteria, designated as strains G1-8^T^ and J1-3^T^, were isolated from estuarine sediment samples collected from Jinjiang Estuary, Quanzhou City, Fujian Province, China. Phylogenetic analysis of 16S rRNA gene and whole-genome sequences, in combination with the genomic relatedness, showed that strains G1-8^T^ and J1-3^T^ represented two novel species within two novel genera of the family *Oleiphilaceae* of the class *Gammaproteobacteria*. Thus, two new names, *Neooleiphilus portus* gen. nov., sp. nov., and *Paroleiphilus aggregans* gen. nov., sp. nov., were proposed based on their phylogenetic relationship with the genus *Oleiphilus*.

**Description of *Neooleiphilus* gen. nov.**

*Neooleiphilus* (Ne.o.o.le.i’phi.lus. Gr. masc. adj. *neos*, new; N.L. masc. n. *Oleiphilus*, a bacterial genus name; N.L. masc. n. *Neooleiphilus*, a new *Oleiphilus*).

Gram-stain-negative, aerobic, mesophilic, neutrophilic and chemoorganotrophic bacteria. Colonies grown on agar plate are small and round. Cellular S-layer structure was observed. Oxidase-positive and catalase-negative. The major fatty acids (>10%) were C_16:0_, C_16:1_ *ω*9*c*, C_18:1_ *ω*9*c*, and summed feature 3. The major isoprenoid quinone is Q-9. The polar lipid included phosphatidylglycerol (PG). It has a closely phylogenetic relationship with *Oleiphilus*. It is affiliated with the family *Oleiphilaceae*, the order *Oceanospirillales* of the class *Gammproteobacteria*. The type species is *Neooleiphilus portus*.

**Description of *Neooleiphilus portus* sp. nov.**

*Neooleiphilus portus* (*por’tus*. L. gen. n. *portus*, of a harbor).

In addition to the genus description, cells are rod-shaped, 1.6–2.9 μm in length and 0.9–1.5 μm in width. Colonies on MB agar plate were small, round, and smooth after incubation for 7 days at 30 °C. Growth is observed at the temperature of 20–40 °C with the optimum of 30 °C, NaCl tolerance of 0–4.0% with the optimum of 1.0–2.0% (*w*/*v*), and pH range of 6.0–8.0 with the optimum of 7.0. Positive for alkaline phosphatase, esterase (C4), esterase lipase (C8), lipase (C14), leucine arylamidase, valine arylamidase acid phosphatase activities, naphthol-AS-BI-phosphohydrolase and arginine dihydrolase, and weakly positive for cystine arylamidase, trypsin, and *α*-chymotrypsinnegative. Nitrate can be reduced into nitrite under aerobic conditions. Hydrolysis of gelatin is positive. Acid can be weakly produced from the D-glucose. The major fatty acids (>10%) are C_16:0_, C_16:1_ *ω*9*c*, C_18:1_ *ω*9*c*, and summed feature 3. The polar lipids consisted of phosphatidylglycerol (PG) and two unidentified phospholipids.

The type strain is G1-8^T^ (=MCCC 1K10098^T^=KCTC 18326^T^), isolated from an estuarine sediment sample collected from Jinjiang Estuary, Quanzhou, Fujian Province, China. The GenBank/EMBL/DDBJ accession number of the 16S rRNA gene sequence and genome sequence of strain G1-8^T^ are PV819317 and JBPJFY000000000, respectively.

**Description of *Paroleiphilus* gen. nov.**

*Paroleiphilus* (Par.o.le.i’phi.lus. Gr. prep. *para*, beside; N.L. masc. n. *Oleiphilus*, a bacterial genus name; N.L. masc. n. *Paroleiphilus*, a genus resembling *Oleiphilus*).

Gram-stain-negative, aerobic, mesophilic, neutrophilic and chemoorganotrophic bacteria. Colonies grown on agar plates are small and round. Cells can aggregate when grown in liquid medium. Oxidase-positive and catalase-negative. The major fatty acids (>10%) were summed feature 3 and C_16:0_. The major isoprenoid quinone is Q-9. The major polar lipid included phosphatidylethanolamine (PE). It has a closely phylogenetic relationship with the genus *Oleiphilus*. It is affiliated with the family *Oleiphilaceae*, the order *Oceanospirillales* of the class *Gammproteobacteria*. The type species is *Paroleiphilus aggregans*.

**Description of *Paroleiphilus aggregans* sp. nov.**

*Paroleiphilus aggregans* (ag’gre.gans. L. fem. part. adj. *aggregans*, assembling, aggregating).

In addition to the genus description, cells are rod-shaped, 1.7–2.2 μm in length and 1.0–1.3 μm in width. Colonies on MB agar plate are small, round, and smooth after incubation for 7 days at 30 °C. Growth is observed at the temperature of 20–40 °C with the optimum of 30 °C, NaCl tolerance of 0–6.0% with the optimum of 1.0–2.0% (*w*/*v*), and pH range of 6.0–8.0 with the optimum of 7.0. Positive for alkaline phosphatase, esterase (C4), esterase lipase (C8), leucine arylamidase, valine arylamidase, acid phosphatase and naphthol-AS-BI-phosphohydrolase, and weakly positive for lipase (C14) and cystine arylamidase. Nitrate can be weakly reduced into nitrite under aerobic conditions. Hydrolysis of aesculin and 4-nitrophenyl-*β*-D-galactopyranoside (PNPG) are positive. The major fatty acids (>10%) are summed feature 3 and C_16:0_. The polar lipids consisted of phosphatidylethanolamine (PE) and two unidentified lipids.

The type strain is J1-3^T^ (=MCCC 1K10092^T^=KCTC 18325^T^), isolated from an estuarine sediment collected from Jinjiang, Quanzhou, Fujian Province, China. The GenBank/EMBL/DDBJ accession number of the 16S rRNA gene sequence and genome sequence of strain J1-3^T^ are PV819316 and JBPJFZ000000000, respectively.

## Figures and Tables

**Table 1:** Differential characteristics between strains G1-8^T^ and J1-3^T^ and their close relatives.

Characteristics	1	2	3 *
An S-layer structure	+	-	-
Growth temperature range (°C)	20–40 (30)	20–40 (30)	10–37 (25–30)
NaCl tolerance range (%, *w*/*v*)	0–6 (1)	0–6 (1–2)	0.06–10.5 (2.5–5)
*α*-chymotrypsin	w	-	ND
Hydrolysis of gelatin	+	-	-
Hydrolysis of aesculin	-	+	-
PNPG	-	+	ND
Reduction of nitrate to nitrite	+	+	+
Arginine dihydrolase	+	-	-
Genome size (bp)	4,948,536	4,888,805	6,379,281
Number of CDS	4388	4376	5545
Genomic G+C content (%)	52.16	46.79	47.80
Polar lipids	PG	PE	PE, PG, DME
Isolation source or habitat	Estuarine Sediment	Estuarine Sediment	Seawater/sediment

* Data were taken from Golyshin et al. 2002 [7]. PNPG, 4-nitrophenyl-β-D-galactopyranoside; ND, no data or not reported. 1. Strain G1-8^T^; 2. strain J1-3^T^; 3. *Oleiphilus messinensis* ME102^T^. +, positive; w, weak positive; -, negative. ND, no data. PNPG, 4-nitrophenyl-*β*-D-galactopyranoside. PE, phosphatidylethanolamine; PG, phosphatidylglycerol; DME, phosphatidyldimethylethylamine.

**Table 2:** The fatty acid composition of strains G1-8^T^ and J1-3^T^ compared with and other close relatives.

Fatty Acid	1	2	3
**Saturated**			
C_12:0_	5.0	4.6	-
C_14:0_	5.5	7.1	5.5
C_16:0_	24.9	37.0	38.6
C_18:0_	2.4	1.2	7.7
**Hydroxy**			
C_12:0_ 3-OH	5.0	3.0	-
**Unsaturated**			
C_16:1_ *ω*9*c*	14.9	-	10.9
C_17:1_ *ω*8*c*	2.0	tr	-
C_18:1_ *ω*9*c*	14.2	tr	1.4
**Summed Features** ^†^			
2	2.4	1.5	-
3	13.1	39.6	16.6
5.	5.2	tr	-

^†^ Summed feature 2 comprised C_12:0_ aldehyde and/or unknown 10.928, summed feature 3 comprised C_16:1_*ω*7*c* and/or C_16:1_ *ω*6*c*, and summed feature 5 comprised C_18:2_ *ω*6,9*c* and/or C_18:0_ ante. 1. Strain G1-8^T^; 2. strain J1-3^T^; 3. *O. messinensis* ME102^T^. All data were obtained from this study or from the reference [7]. -, not detected or not reported; tr, trace < 1%.

## Data Availability

The 16S rRNA gene sequences of strain G1-8^T^ and strain J1-3^T^ have been deposited in DDBJ/ENA/GenBank under the accession numbers PV819317 and PV819316, respectively. The whole-genome sequences of strain G1-8^T^ and strain J1-3^T^ have been deposited in DDBJ/ENA/GenBank under the accession numbers JBPJFY000000000 and JBPJFZ000000000, respectively.

## Supplementary Materials

The following supporting information can be downloaded at https://www.mdpi.com/article/10.3390/microorganisms14102307/s1. Figure S1. Satellite map showing the two sampling sites (G and J) alongside the Jinjiang estuary in Quanzhou City, Fujian Province, China. Figure S2. The maximum-likelihood phylogenetic tree constructed using 16S rRNA gene sequences showing the relationship between strains G1-8T and J1-3T and the close relatives of the family Oleiphilaceae. Figure S3. Heatmap of average amino acid identity (AAI) values showing the clear difference between the two strains G1-8T and J1-3T. Figure S4. The polar lipid profiles of strain G1-8T (left panel) and strain J1-3T (right panel). Table S1. Genomic relatedness compared among the genomes. Table S2. Functional genes predicted from the genomes of strains G1-8T and J1-3T.

## Abbreviations

MCCC	Marine Culture Collection of China
KCTC	Korean Collection for Type Cultures
dDDH	Digital DNA-DNA hybridization
ANI	Average nucleotide identity
AAI	Average amino acid identity
