# Supplementary material for: Neooleiphilus portus gen. nov., sp. nov. and Paroleiphilus aggregans gen. nov., sp. nov., Two Novel Nitrate-Reducing Bacteria Isolated from Estuarine Sediments
Source: Microorganisms. 2026 Oct 10;14(10):2307. doi: 10.3390/microorganisms14102307 (PMC13707976; doi:10.3390/microorganisms14102307)
Supplement: Supplementary file 1 [file microorganisms-14-02307-s001.zip › microorganisms-4541727-supplementary.pdf]

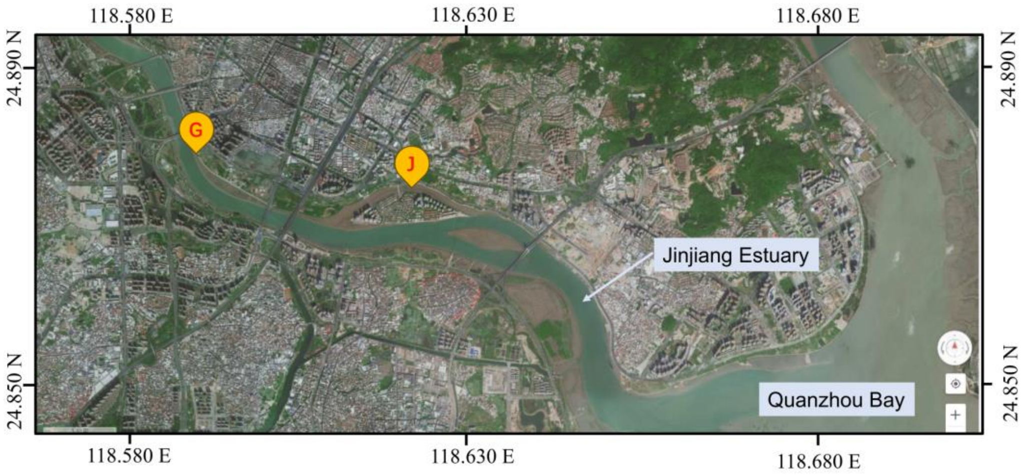

Figure S1. Satellite map showing the two sampling sites (G and J) alongside the Jinjiang estuary in Quanzhou City, Fujian Province, China.

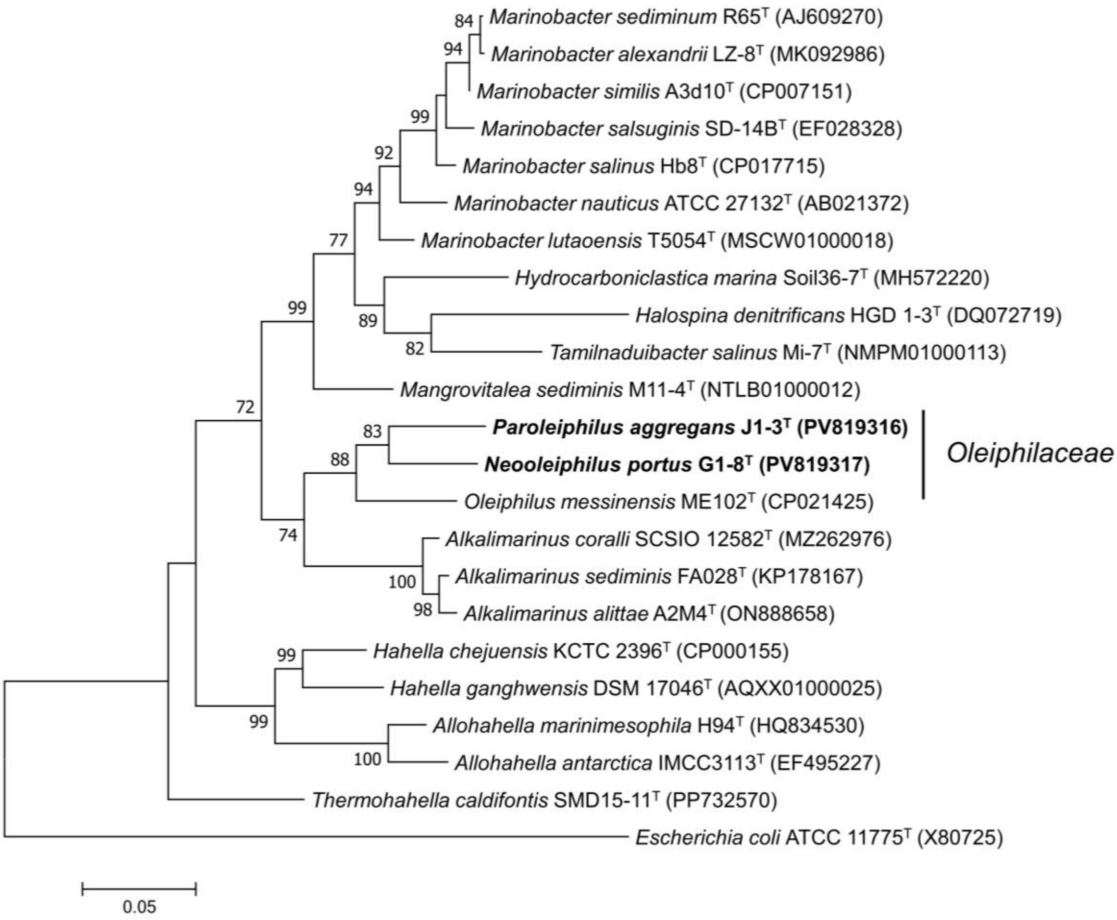

Figure S2. The maximum-likelihood phylogenetic tree constructed using 16S rRNA gene

sequences showing the relationship between strains G1-8^T and J1-3^T and the close relatives of the family *Oleiphilaceae*. Bootstrapping was carried out with 1,000 replicates. The node values below 70% are not shown on the branch. Bar, 0.05 represented the nucleotide substitution per position.

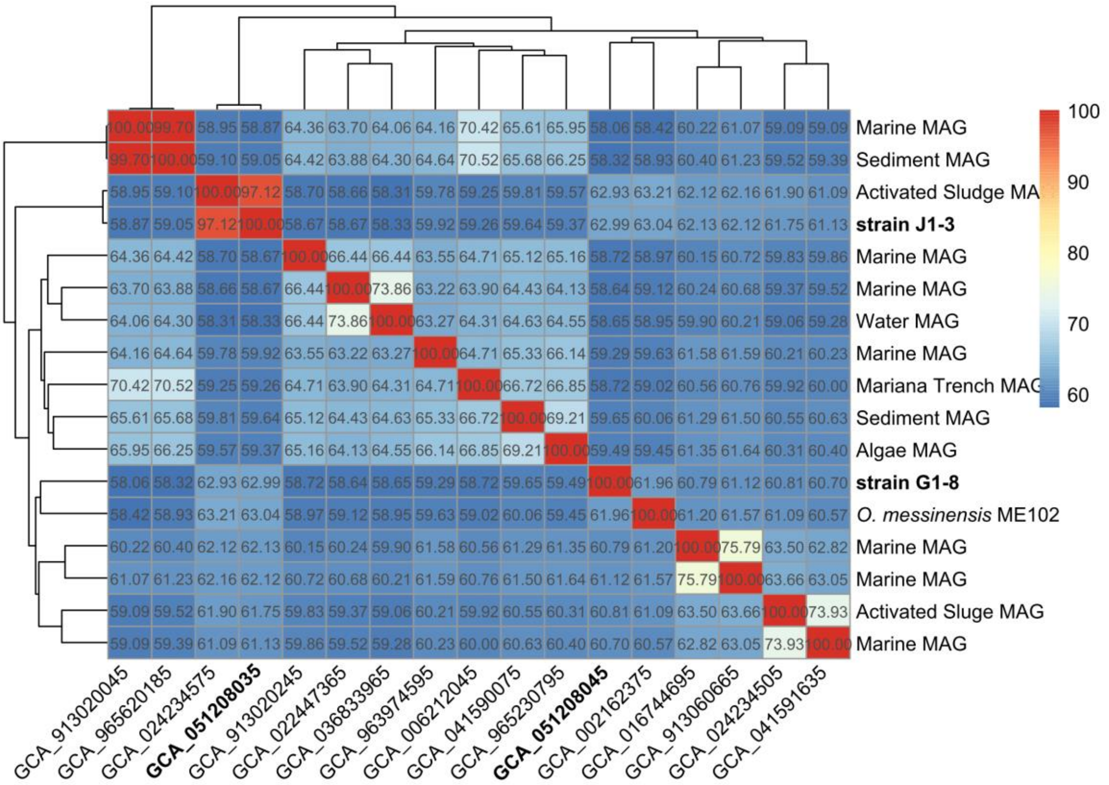

Figure S3. Heatmap of average amino acid identity (AAI) values showing the clear difference between the two strains G1-8^T and J1-3^T.

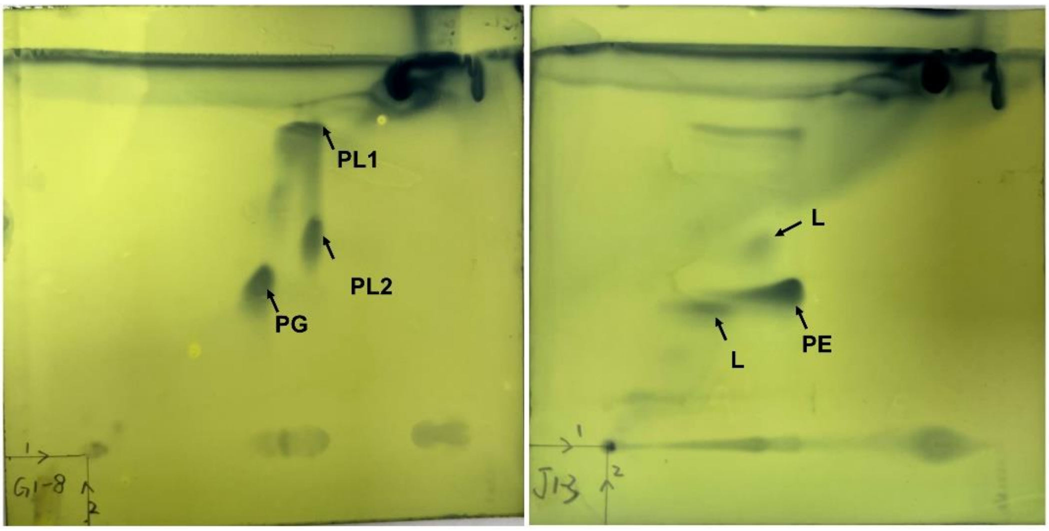

Figure S4. The polar lipid profiles of strain G1-8^T (left panel) and strain J1-3^T (right panel). The total lipid was detected using molybdatophosphoric acid. PG, phosphatidylglycerol, PE, phosphatidylethanolamine, PL, phospholipids, L, unidentified lipid.

Table S1. Genomic relatedness compared among the genomes.

Strain	Strain or MAG*	dDDH	ANI
G1-8^T	J1-3^T	17.9	68.2
G1-8^T	*O. messinensis* strain ME102^T	22.6	68.6
J1-3^T	*O. messinensis* strain ME102^T	21.2	68.8
G1-8^T	*A. sediminis* strain FA028^T	20.5	67.8
J1-3^T	*A. sediminis* strain FA028^T	22.1	68.1
J1-3^T	HK-STAS-PROT-159*	66.9	96.3

*MAG, metagenome-assembled genome.

Table S2. Functional genes predicted from the genomes of strains G1-8^{T} and J1-3^{T}.

Strains	Genes	Description	eggNOG_OGs	KEGG_ko
G1-8	*nar*I	nitrate reductase, gamma subunit	COG2181	K00374
	*nar*J	Nitrate reductase delta subunit	COG2180	K00373
	*nar*H	Nitrate reductase beta subunit	COG1140	K00371
	*nar*G	Belongs to the prokaryotic molybdopterin-containing oxidoreductase family	COG5013	K00370
	*nar*K	PFAM major facilitator superfamily MFS_1	COG2223	K02575
J1-3	*nap*D	periplasmic nitrate reductase	COG3062	K02570
	*nap*A	nitrate reductase (NAP)	COG0243	K02567
	*nap*G	Ferredoxin-type protein	COG0437	K02573
	*nap*H	NapH MauN family	COG0348	K02574
	*nap*B	periplasmic nitrate reductase complex NapAB	COG3043	K02568
ME102	*nap*D	periplasmic nitrate reductase	COG3062	K02570
	*nap*A	nitrate reductase (NAP)	COG0243	K02567
	*nap*B	periplasmic nitrate reductase complex NapAB	COG3043	K02568
